# Folic Acid–Functionalized Composite Scaffolds of Gelatin and Gold Nanoparticles for Photothermal Ablation of Breast Cancer Cells

**DOI:** 10.3389/fbioe.2020.589905

**Published:** 2020-11-04

**Authors:** Huajian Chen, Xiuhui Wang, Linawati Sutrisno, Tianjiao Zeng, Naoki Kawazoe, Yingnan Yang, Guoping Chen

**Affiliations:** ^1^Research Center for Functional Materials, National Institute for Materials Science, Tsukuba, Japan; ^2^Department of Materials Science and Engineering, Graduate School of Pure and Applied Sciences, University of Tsukuba, Tsukuba, Japan; ^3^Institute of Translational Medicine, Shanghai University, Shanghai, China; ^4^Graduate School of Life and Environmental Science, University of Tsukuba, Tsukuba, Japan

**Keywords:** photothermal therapy, folic acid, composite scaffolds, gold nanoparticles, cell targeting

## Abstract

Photothermal therapy (PTT) has been developed as a useful therapeutic method for cancer treatment. Localization of PTT agents in cancer sites and targeting capacity are required to further increase therapeutic efficacy. In this study, gold nanoparticles (AuNPs) and gelatin were functionalized with folic acid (FA) and hybridized to prepare FA-functionalized gelatin–AuNPs composite scaffolds. AuNPs with rod and star shapes of three sizes (40, 70, and 110 nm) were used for the hybridization to investigate the influence of AuNPs shape and size. The composite scaffolds showed porous structures with good interconnectivity. Modification with FA increased capture capacity of the composite scaffolds. Hybridization with AuNPs rendered the composite scaffold a good photothermal conversion property under near-infrared (NIR) laser irradiation. Temperature change during laser irradiation increased with the laser power intensity and irradiation time. The shape and size of AuNPs also affected their photothermal conversion property. The composite scaffold of gold nanorods 70 (FA-G/R70) had the highest photothermal conversion capacity. Breast cancer cells cultured in the FA-G/R70 composite scaffold were killed under NIR laser irradiation. Mouse subcutaneous implantation further demonstrated the excellent photothermal ablation capability of FA-G/R70 composite scaffold to breast cancer cells. The FA-functionalized composite scaffolds were demonstrated a high potential for local PPT of breast cancer.

## Introduction

Breast cancer has high incident and mortality ratio and is a serious threat to human health (Bray et al., 2018; Ji et al., 2020). Surgery has widely used to cure breast cancer patients (Waks and Winer, 2019). However, complete elimination and prevention of local recurrence remain problematic for surgical resection of breast cancer (Laurberg et al., 2017; Niinikoski et al., 2019; Li et al., 2020). In recent years, many other therapeutic methods such as immunotherapy, gene therapy, photothermal therapy (PTT), and photodynamic therapy have been developed to overcome the limitations of surgical resection (Bai et al., 2019; Nassa et al., 2019; Feng et al., 2020). Among them, PTT, which converts near-infrared (NIR) laser into hyperthermal effect for ablating cancer cells, has attracted broad attention due to its highly therapeutic effect (Kim et al., 2006; Li et al., 2015; Chen M.C. et al., 2016). A critical factor that realizes photothermal conversion is photothermal agents (Vines et al., 2019). Currently, various photothermal agents (PTT agents) have been studied. They include copper sulfide nanoparticles (Wang et al., 2015), carbon nanoparticles (Han et al., 2018), and gold nanoparticles (AuNPs) (Liu et al., 2015; Cheng et al., 2017; Alamzadeh et al., 2019; Wang et al., 2019a). AuNPs have been recognized as one of the most promising photothermal agents because of their good biocompatibility and high photothermal conversion efficiency (Deng et al., 2014). They have another advantage of tunable NIR absorbance due to their localized surface plasmon resonance (LSPR) (Ding et al., 2014; Eyvazzadeh et al., 2017), which means photothermal conversion capability of AuNPs can be controlled by adjusting their shapes and sizes (Hwang et al., 2014). Benefited from these features, optimal cancer therapeutic effect can be achieved by choosing the appropriate AuNPs (Mackey et al., 2014; Moustaoui et al., 2019).

Successful delivery of photothermal agents is important to realize photothermal therapeutic effect. AuNPs can be delivered through intravenous injection of free AuNPs or implantation with a combination of scaffolds (Blanco et al., 2015; Li et al., 2018; Wang et al., 2018, 2019b). Intravenous injection of free AuNPs has the problems of immunological clearance by host responses and unexpected accumulation in healthy tissues and organs (Attarilar et al., 2020). Recently, implantation of composite scaffolds of nanoparticles and biocompatible polymers or bioceramics has been reported to directly deliver PTT nanoparticles to the cancer tissues or the resected sites (Eivazzadeh-Keihan et al., 2019; Luo et al., 2019; Chen et al., 2020). The composite scaffolds can not only restrict unexpected diffusion of free nanoparticles, but also exhibit high local heating effect and repeat ablation effect (Abdelrasoul et al., 2015; Escudero-Duch et al., 2019). Furthermore, the composite scaffolds can have another function to induce regeneration of resected tissues (Chen S. et al., 2016). Except localization of nanoparticles in the expected sites, specific or selective capability to capture cancer cells is required to further increase the ablation efficiency. When the composite scaffolds are degraded, the nanoparticles are released and can be expected to be uptaken by cancer cells for long-term photothermal ablation. Targeting capacity of the released nanoparticles is required to further improve ablation efficiency. To achieve these effects, composite scaffolds and nanoparticles have been modified with ligands that have high affinity to their receptors on the surfaces of cancer cells (Li et al., 2015; Zhang et al., 2017b). Folic acid (FA) has been frequently used as targeting ligand in nanoparticles (Khademi et al., 2018; Montazerabadi et al., 2019), liposomes, and drugs to increase their targeting capacity. FA can specifically recognize and bind the FA receptors overexpressed by breast cancer cells (Li et al., 2017; He et al., 2019, 2020).

Therefore, in this study, AuNPs were hybridized with gelatin, which is biocompatible and broadly used for biomedical applications, to prepare composite scaffolds of AuNPs and gelatin. FA was bound to both gelatin and AuNPs to provide the composite scaffolds with targeting capacity to breast cancer cells. The composite scaffolds should have captured capacity for FA receptor–expressing breast cancer cells. Meanwhile, the released FA-modified AuNPs after scaffold degradation should have targeting capacity for breast cancer cells. The porous structure of the composite scaffolds was controlled by using pre-prepared ice particulates as a porogen material. The FA-functionalized gelatin–AuNPs composite scaffolds were used for culture of breast cancer cells. The targeting capacity and *in vitro* and *in vivo* photothermal ablation effects of the composite scaffolds were investigated.

## Materials and Methods

### Materials

Folic acid, dimethyl sulfoxide (DMSO), 6 mol ⋅ L^–1^ hydrochloric acid (HCl) and sodium hydroxide, ethanol, acetic acid, trisodium citrate dihydrate, glycine, hydroquinone, hydrogen tetrachloroaurate tetrahydrate (HAuCl_4_4H_2_O, 99.9%), dicyclohexylcarbodiimide (DCC), and *N*-hydroxysuccinimide (NHS) were purchased from Wako Pure Industries, Ltd., (Tokyo, Japan). Hexadecyltrimethylammonium bromide (CTAB), 2-(*N*-morpholino)ethanesulfonic acid (MES), Dulbecco modified eagle medium (DMEM), penicillin, streptomycin, L-glutamine, trypsin/EDTA, ascorbic acid (AA), sodium borohydride (NaBH_4_), and silver nitrate (AgNO_3_) were purchased from Sigma–Aldrich (St. Louis, MO, United States). Porcine gelatin was provided by Nitta Gelatin (Osaka, Japan). Collagenase type I was purchased from Worthington Biochemical Corporation (Lakewood, NJ, United States). All chemicals were used without further purification.

### Synthesis of Folic Acid–Conjugated Gelatin

FA-conjugated gelatin was synthesized by activating carboxyl groups of FA and reacting with amino groups of the gelatin. At first, FA was activated to FA-NHS. Four hundred milligrams of FA was dissolved in 10 mL DMSO and mixed with 374 mg DCC dissolved in 10 mL DMSO and 84 mg NHS dissolved in 20 mL DMSO. The mixture was kept stirring for 6 h at room temperature to activate the carboxyl groups of FA. After activation, the mixture was centrifuged (12,000 rpm, 30 min) to remove extra DCU. The activated FA-NHS was precipitated by adding diethyl ether and acetone mixture solution (70:30, vol/vol) into the supernatant at −20°C and centrifuged (12,000 rpm, 10 min) at −2°C. The products were resolved in DMSO and repeated above two steps twice before dried in vacuum. Subsequently, the FA-NHS was dissolved in DMSO and added to gelatin aqueous solution that was prepared by dissolving gelatin in sodium carbonate buffer solution. The mixture solution was kept stirring overnight at room temperature. Finally, the mixture solution was dialyzed with DMSO–water mixture solvent having different volume ratio of DMSO and pure water (80:20, 70:30, 60:40, vol/vol) and pure water only (dialysis membrane with a molecular weight cutoff of 3.5 kDa). FA-modified gelatin was obtained after lyophilization and stored at 4°C for following use.

### Preparation of Folic Acid–Coated AuNPs With Different Shapes and Sizes

Au nanorods (AuNRs) with different sizes (around 40, 70, and 110 nm in length) and Au nanostars (AuNSs) with different sizes (around 40, 70, and 110 nm) were prepared by previously reported method (Zhang et al., 2017a). Briefly, the AuNRs (40 and 70 nm) were prepared by seed-growth method. With stabilization by CTAB, HAuCl_4_ was quickly reduced by NaBH_4_ and became Au seed. The AuNR40 (40 nm) and AuNR70 (70 nm) were prepared by adjusting the amount of AgNO_3_ (0.44 and 2.20 mL of 10 mM AgNO_3_, respectively) in the growth solution. The growth solution contained 10 mL of 0.01 M HAuCl_4_, 4 mL of 1 M HCl, 1.6 mL of 0.1 M AA, 0.48 mL of Au seed, and 150 mL of 0.1 M CTAB. The AuNR110 (110 nm) was prepared by a one-pot method. Briefly, 7.6 mL of 0.01 M HAuCl_4_, 2 mL of 0.02 M AgNO_3_, and 1.6 mL of 0.62 M hydroquinone were mixed with 178 mL of 0.11 M CTAB solution. And then, 126 μL of 0.5 M NaBH_4_ was added and the mixture was kept at 30°C for 24 h to obtain AuNR110. The AuNSs were also synthesized by seed-growth method with a little change of the aforementioned conditions for AuNRs preparation. The seed solution for AuNSs was prepared by using trisodium citrate to reduce HAuCl_4_. The growth solution of AuNSs (40, 70, and 110 nm) consisted of 0.75 mL of 30 mg ⋅ mL^–1^ HAuCl_4_, 0.3 mL of 5 mg ⋅ mL^–1^ AgNO_3_, and 0.5 mL of 38 mg ⋅ mL^–1^ AA and Au seed (25, 5, and 1.1 mL, respectively). After AuNRs and AuNSs were prepared, they were mixed with 0.5% (wt/vol) of FA–gelatin solution and stirring at room temperature for 24 h. The FA-functionalized AuNPs were collected by centrifugation, washed with Milli-Q water, and redispersed in Milli-Q water for further use.

### Preparation of FA-Functionalized Gelatin–AuNPs Composite Scaffolds

In order to control the porous structure of composite scaffolds, pre-prepared ice particulates were used as a porogen material as previously reported (Zhang et al., 2013). At first, ice particulates were prepared by spaying Milli-Q water into liquid nitrogen and sieving through mesh sieves with pore size ranging between 425 and 500 μm. Subsequently, the aqueous solution of FA-functionalized AuNPs was dropped into 8% (wt/vol) of FA-conjugated gelatin solution containing 70% of acetic acid. Seventy percent of acetic acid solution could protect freezing of the mixture solution during mixing with ice particulates. The final concentration of AuNPs was 2.0 mM. And then, the ice particulates were added to the FA–gelatin/AuNPs mixture solution at a ratio of 7:3 (wt/vol). The ice particulates/FA–gelatin/AuNPs mixture solution was added to a silicone mold at −4°C, followed with freezing at −20°C for 12 h and −80°C for 4 h. Finally, the frozen constructs were freeze-dried. After freeze-drying, the constructs were washed with ethanol and cross-linked with 50.0 mM EDC and 20.0 mM NHS dissolved in a mixture solution of ethanol and 0.1% (wt/vol) MES mixture solution at a series of ethanol–water ratio of 95/5, 90/10, and 85/5 (vol/vol), each for 8 h. After cross-linking, the composite scaffolds were washed with Milli-Q water for six times and immersed in 0.1 M glycine aqueous solution for 6 h to block the activated groups that were not reacted during cross-lining. The composite scaffolds were washed by Milli-Q water for six times and freeze-dried. Scaffolds of FA-functionalized gelatin, pristine gelatin without AuNPs, and pristine gelatin with bare AuR70 were prepared as controls by the same method described above. These scaffolds were denoted as FA-G (control), G (control), G/R70, FA-G/R40, FA-G/R70, FA-G/R110, FA-G/S40, FA-G/S70, and FA-G/S110, respectively.

### Characterization of AuNPs and FA-Functionalized Gelatin–AuNPs Composite Scaffolds

Transmission electron microscopy (TEM; JEOL 2011F, Japan) was used for observation of AuNPs. An ImageJ software (National Institutes of Health, Bethesda, MD, United States) was used to measure the longitudinal length and transverse length of AuNRs and the distance between the two most distal points of AuNSs. Scanning electron microscopy (SEM; Hitachi S-4800, Japan) was used for observation of the porous structures of the composite scaffolds. UV-visible spectra of all the samples were measured by a UV-2600 UV-visible spectrophotometer (Shimadzu Corp., Japan).

### Quantification of FA Amount in FA-Functionalized Gelatin–AuNPs Composite Scaffolds

Folic acid amount in each scaffold was measured by using a previously reported method (Escudero-Duch et al., 2019). At first, discs (φ 6 mm × H 1 mm) of the FA-functionalized gelatin–AuNPs composite scaffolds were dissolved in 2 mL of 6 M HCl solution for 24 h. And then, the solutions were centrifuged for excluding the influence of AuNPs, and pH of the solutions was adjusted to 13. Finally, UV-visible spectrophotometer was used to measure the absorbance of the supernatants at a wavelength of 365 nm, which was the specific absorbance peak of FA. FA concentration was calculated according to a standard curve. Every three samples were used for the measurement.

### Cell Capture Experiment

MDA-MB231 breast cancer cells that express FA receptors on their membrane were used as FA receptor–positive cells for cell capture experiment (Soe et al., 2018). HT1080 cells that are FA receptor–negative cell line were used as a negative control (Stefflova et al., 2007). The two types of cells were subcultured in cell culture polystyrene flasks with DMEM supplemented with 10% fetal bovine serum, streptomycin (100 μg ⋅ mL^–1^), and penicillin (100 U ⋅ mL^–1^). The confluent cells were detached by treatment with 0.05% trypsin-EDTA, washed once with phosphate-buffered saline (PBS), and resuspended in serum-free DMEM at a concentration of 2 × 10^5^ cells/mL. Cell capture experiment was conducted in a flowing chamber that consisted of a silicone frame on a cell strainer. At first, a sterile silicone frame with a hole (φ 6 mm) was placed on a cell strainer (pore size was 70 μm). The sterilized scaffold discs (φ 6 mm × H 1 mm) were placed in the frame hole and on the cell strainer. The scaffold discs were pre-wetted with PBS and washed with serum-free DMEM by dropping medium on the scaffold discs. And then, the cell suspension solution in serum-free medium was added dropwise on the top of scaffold discs at a speed of 5 s per drop. After 5 mL (1 × 10^6^ cells) of the cell solution was added, 30 μL of serum-free DMEM was added for three times to remove unadhered cells. Finally, the scaffold discs were digested with collagenase type I solution, and cell number in each scaffold disc was counted. Four types of scaffolds (G, G/R70, FA-G, FA-G/R70) were used for cell capture experiment. Every three samples were used for the measurement to calculate means and standard deviations.

### *In vitro* Photothermal Property and Ablation Capacity of FA-Functionalized Gelatin–AuNPs Composite Scaffolds

The G, G/R70, and FA-G/R70 scaffolds were cut into discs (φ 6 mm × H 1 mm) and pregnant with 30 μL Milli-Q water. The hydrated scaffold discs were irradiated with 805-nm laser of different power intensities (1.3, 1.4, 1.5, and 1.6 W/cm^2^) for 10 min, and temperature of the hydrated scaffold discs was recorded each 30 s by electronic thermometer during the irradiation. The laser beam spot size was 8 × 6 mm. For investigation of *in vitro* PTT ablation capacity, the scaffold discs were sterilized with 70% ethanol and washed with PBS for three times. DMEM serum medium was used for cell culture; 20 μL cell suspension solution (5 × 10^6^ cells/mL) of MDA-MB231 cells was seeded on one side of the scaffold discs and cultured in DMEM serum medium for 6 h. After 6 h, the scaffold discs were turned upside down, and another side was seeded with another 20 μL cell suspension solution (5 × 10^6^ cells/mL). After cell seeding, the cell/scaffold constructs were transferred into culture dish and incubated in DMEM serum medium for 1 day. And then, the cell/scaffold constructs were irradiated with 805-nm laser of two power intensities (1.3 and 1.6 W/cm^2^) for 3 and 6 min. MDA-MB231 cells were also seeded and cultured in the FA-functionalized gelatin without AuNPs (control) and irradiated with NIR laser as described above. After NIR laser irradiation, cell/scaffold constructs were continuously cultured for 3 h. And then, cell viability was measured by WST-1 assay. A microplate reader (Benchmark Plus; Bio-Rad, Hercules, CA, United States) was used to measure the absorbance at 440 nm. Live/dead staining kit was used to visualize live and dead cells in the scaffolds with or without laser irradiation. The cell/scaffold constructs with or without NIR laser irradiation were washed by PBS and staining by calcein AM and propidium iodide in serum-free medium for 15 min. The stained cells were observed with a fluorescence microscope (Olympus, Japan).

### *In vivo* Photothermal Ablation Capacity of FA-Functionalized Gelatin–AuNPs Composite Scaffolds

The MDA-MB231-Luc cells were seeded in the G, G/R70, and FA-G/R70 scaffolds (φ 6 mm × H 1 mm) as described above and cultured in DMEM serum medium for 3 day. And then, the cell/scaffold constructs were subcutaneously implanted in 6-week-old female BALB/c nude mice. After implantation for 3 day, the implanted sites were irradiated with 805-nm laser at a power intensity of 1.6 W/cm^2^ for 10 min, and the temperature change was detected by an infrared thermal imager. In order to evaluate PTT ablation capacity, an *in vivo* vision system (IVIS Lumina II, Japan) was used for detecting systemic bioluminescence of MDA-MB231-Luc cells. L-Luciferase solution was administered by intraperitoneal injection the next day after laser irradiation. After injection for 10 min, the IVIS imaging was used to detect the bioluminescence. Every three mice were used for each scaffold group. Systemic bioluminescence was examined before and after laser irradiation for each mouse. The animal experiment procedures were approved by the Animal Experiments Committee of National Institute for Materials Science, and the experiments were conducted according to the committee guidelines.

### Statistical Analysis

All the quantitative experiments were repeated in triplicate (*n* = 3). All the results were expressed as mean ± standard deviation. The statistical analysis of experimental data was performed by using one-way analysis of variance statistical analysis. The *p* value was used to determine the level of significance: ^∗^*P* < 0.05, ^∗∗^*P* < 0.01, and ^∗∗∗^*P* < 0.001.

## Results and Discussion

### Characterization of AuNRs and AuNSs

**FIGURE 1:**
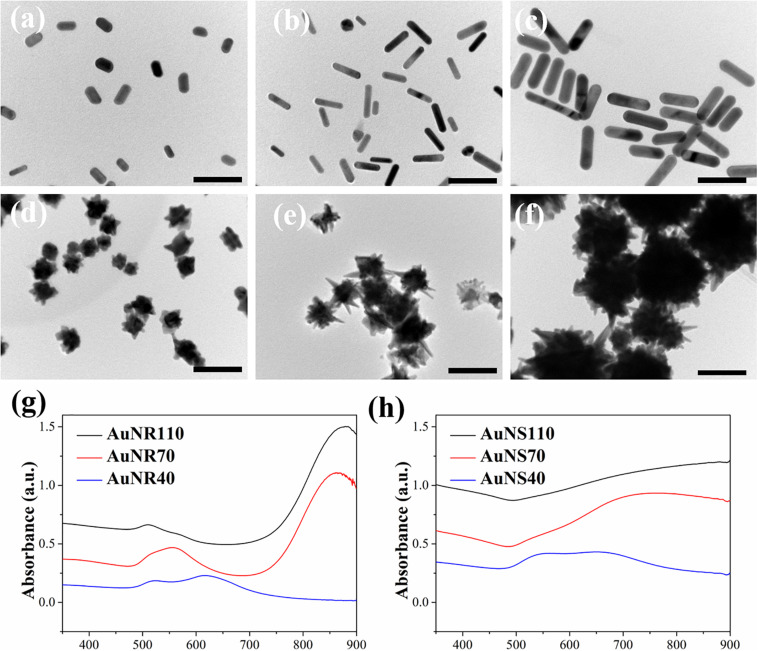
TEM images of AuNPs: **(a)** AuNR40, **(b)** AuNR70, **(c)** AuNR110, **(d)** AuNS40, **(e)** AuNS70, **(f)** AuNS110. Scale bar: 100 nm. UV-visible spectra of AuNRs **(g)** and AuNSs **(h)**.

Transmission electron microscopy images showed the rod- and star-shaped morphology of the AuNRs (Figures 1a–f). The AuNR40, 70, and 110 had a length × width dimension of (39.6 ± 5.7 nm) × (19.0 ± 2.6 nm), (68.3 ± 4.7 nm) × (14.2 ± 2.1 nm) and (113.8 ± 13.8 nm) × (25.6 ± 3.8 nm), respectively. The AuNS40, 70, and 110 had a dimension of 37.1 ± 2.9, 68.5 ± 10.2, and 113.9 ± 7.2 nm, respectively. UV-visible spectra of the AuNRs and AuNSs showed that the AuNR70 and AuNR110 had an absorbance peak at 833 and 880 nm, respectively. AuNR40 had an absorbance peak at 611 nm. AuNSs showed a broad absorbance peak around 800 nm. The difference of UV-visible absorbance spectra and absorbance peaks among these AuNRs and AuNRs could be attributed to LSPR effect of AuNPs (Zhao et al., 2009). By comparing the UV-visible spectra of all these AuNPs, AuNR70 had a relatively sharp and high absorbance peak in NIR area, suggesting AuNR70 should have the highest absorbance capability under NIR laser irradiation.

### Preparation and Characterization of FA-Functionalized Gelatin–AuNPs Composite Scaffolds

UV-visible spectrum was used to confirm conjugation between FA and gelatin (Hao et al., 2018). Gelatin had no absorbance peak between wavelength of 300 and 900 nm, whereas FA had a sharp and strong absorbance peak at a wavelength of 365 nm (Supplementary Figure S1). FA–gelatin showed a broad absorbance around 365 nm. The results indicated that FA was conjugated with gelatin. FA amount in the FA-functionalized gelatin measured at 365 nm was 46.8 ± 5.1 μg/mg. UV-visible spectrum was also used for analyzing absorbance change of AuNR70 before and after modification with FA–gelatin (Mehdizadeh et al., 2014). FA–gelatin–coated AuNR70 showed an increased absorbance peak around 365 nm, suggesting modification of AuNR70 with FA (Supplementary Figure S2).

**FIGURE 2:**
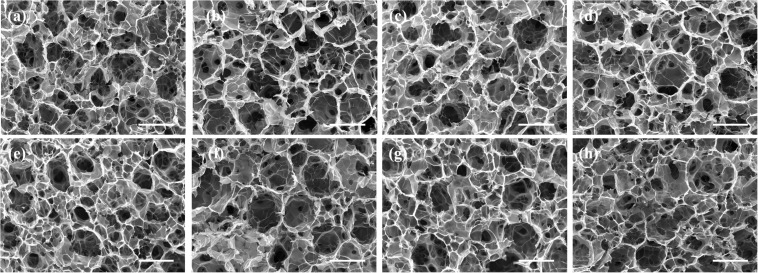
SEM images of FA-functionalized gelatin–AuNPs composite scaffolds and FA-functionalized gelatin and gelatin control scaffold: **(a)** G (gelatin scaffold), **(b)** FA-G (FA-functionalized gelatin scaffold), **(c)** FA-G/R40, **(d)** FA-G/R70, **(e)** FA-G/R110, **(f)** FA-G/S40, **(g)** FA-G/S70, **(h)** FA-G/S110. Scale bar: 500 μm.

The FA-functionalized gelatin–AuNPs composite scaffolds were prepared by hybridizing FA–gelatin-coated AuNR40, 70, and 110 and AuNS40, 70, and 110 with FA-functionalized gelatin porous scaffolds, respectively. Pre-prepared ice particulates with a diameter between 425 and 500 μm were used as a porogen material to control the porous structures. Gelatin and FA-functionalized gelatin porous scaffolds were prepared as controls by the same method. Porous structures of the scaffolds were observed by SEM (Figure 2). All the scaffolds showed similar porous structures. There were many large spherical pores that were surrounded with small pores. The large spherical pores had identical sizes to the ice particulates (∼425–500 μm) and were well interconnected. The interconnected pores ensured that seeded cells could penetrate and distribute throughout the whole scaffold (Zhang et al., 2014).

In order to quantify the amount of FA in each composite scaffold, the scaffolds were dissolved in HCl solution, and absorbance of the solutions at 365 nm was measured to calculate FA amount. The FA amount in FA-G/R40, FA-G/R70, FA-G/R110, FA-G/S40, FA-G/S70, and FA-G/S110 was 47.3 ± 5.3, 46.8 ± 8.4, 47.4 ± 6.2, 48.4 ± 6.5, 50.3 ± 9.7, and 49.5 ± 7.5 μg/mg, respectively. FA amount was almost at the same levels for all the composite scaffolds because the same FA-conjugated gelatin was used for the preparation of all the composite scaffolds. The FA-functionalized AuNPs showed no evident influence on FA amount in the composite scaffolds.

### Photothermal Conversion Property of FA-Functionalized Gelatin–AuNPs Composite Scaffolds

**FIGURE 3:**
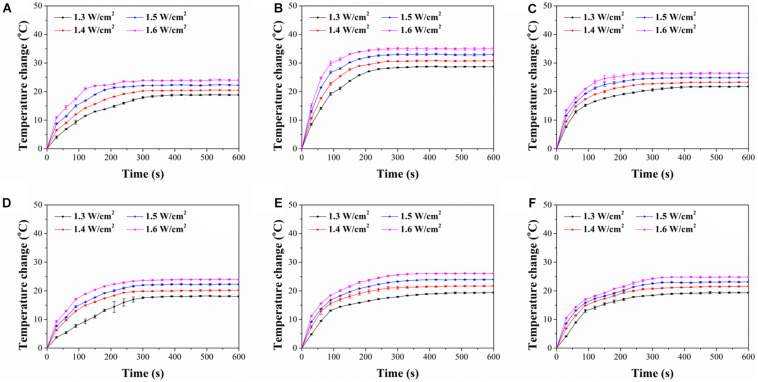
Time course of temperature change of composite scaffolds during NIR laser irradiation with different laser power intensities of 1.3, 1.4, 1.5, and 1.6 W/cm^2^ for 600 s: **(A)** FA-G/R40, **(B)** FA-G/R70, **(C)** FA-G/R110, **(D)** FA-G/S40, **(E)** FA-G/S70, **(F)** FA-G/S110.

The photothermal conversion property of FA-functionalized gelatin–AuNPs composite scaffolds was investigated by irradiating the scaffolds with 805-nm laser at power intensity of 1.3, 1.4, 1.5, and 1.6 W/cm^2^ (Figure 3). The results indicated that all composite scaffolds showed rapid temperature increase during the first 200-s NIR laser irradiation and then reached temperature plateau afterward. The magnitude of temperature change increased with NIR laser intensity. The six types of composite scaffolds had different magnitude of temperature change (Table 1). Among the composite scaffolds, FA-G/R70 showed the highest photothermal conversion property. The different photothermal conversion property should be attributed to the different absorbance capacity AuNPs under NIR laser irradiation as shown in Figure 1. The shape and size of AuNPs can LSPR absorption (Liu et al., 2011), which results in shift of UV-visible absorbance. The absorbance peak of AuNR70 was closest to the wavelength of NIR laser, and the sharp absorbance peak indicated specific absorbance to the NIR laser. Therefore, the composite scaffold FA-G/R70 was used for the following *in vitro* and *in vivo* cell culture experiments.

**TABLE 1:** Temperature change (°C) of FA-G and FA-G/AuNPs composite scaffolds after 10-min laser irradiation. Data represent mean ± SD.

	**1.3 W/cm^2^**	**1.4 W/cm^2^**	**1.5 W/cm^2^**	**1.6 W/cm^2^**
FA-G	4.60.2	5.80.1	7.60.2	9.00.5
FA-G/R40	18.80.2	20.40.1	22.30.2	23.90.3
FA-G/R70	28.70.1	30.80.2	33.00.3	35.10.4
FA-G/R110	21.80.1	23.30.1	24.90.5	26.30.3
FA-G/S40	18.10.3	20.10.2	22.20.3	24.00.2
FA-G/S70	19.40.3	21.70.2	24.00.1	26.10.1
FA-G/S110	19.30.1	21.60.2	23.10.2	25.00.1

### Capture Capability of Cancer Cell by FA-Functionalized Gelatin–AuNPs Composite Scaffolds

**FIGURE 4:**
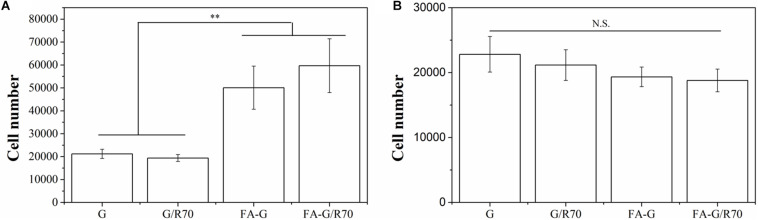
Captured number of MDA-MB231 cells (FA receptor–positive cells) and HT1080 cells (FA receptor–negative cells) in G, G/R70, FA-G, and FA-G/R70 scaffolds: **(A)** MDA-MB231 cells, **(B)** HT1080. Data represent means ± SD (*n* = 3). **P < 0.01; N.S., no significant difference.

Instead of static cell culture in scaffolds (Escudero-Duch et al., 2019), a flowing cell capture method was used to evaluate cell capture capacity of the composite scaffolds. Two types, MDA-MB231 cells (FA receptor–positive cells) and HT1080 cells (FA receptor–negative cells), were used for cell culture to investigate the specific capture property of FA-functionalized composite scaffolds for FA receptor–positive cancer cells. The FA-functionalized composite scaffolds (FA-G/R70) and FA-functionalized gelatin (FA-G) captured significantly more MDA-MB231 cells than did the non-functionalized composite scaffold (G/R70) and gelatin scaffold (G) (Figure 4A). The number of captured MDA-MB231 cells in FA-G/R70 and FA-G had no significant difference. There was either no significant difference of captured number of MDA-MB231 cells in G/R70 and gelatin scaffolds. However, when HT1080 cells were cultured in the scaffolds, the number of captured cells in all the scaffolds had no significant difference (Figure 4B). The results indicated that the FA receptor–positive breast cancer cells could be efficiently captured by the FA-functionalized scaffolds. This should be due to the specific interaction between the FA molecules in the scaffolds and FA receptors on the cell membrane of MDA-MB231 cells. The incorporated AuNR70 in the composite scaffolds did not affect the cell capture capacity of the composite scaffolds because the AuNPs were coated with FA-conjugated gelatin and embedded in the pore walls of the scaffolds.

### *In vitro* Photothermal Ablation of Cancer Cells by FA-Functionalized Gelatin–AuNPs Composite Scaffolds

**FIGURE 5:**
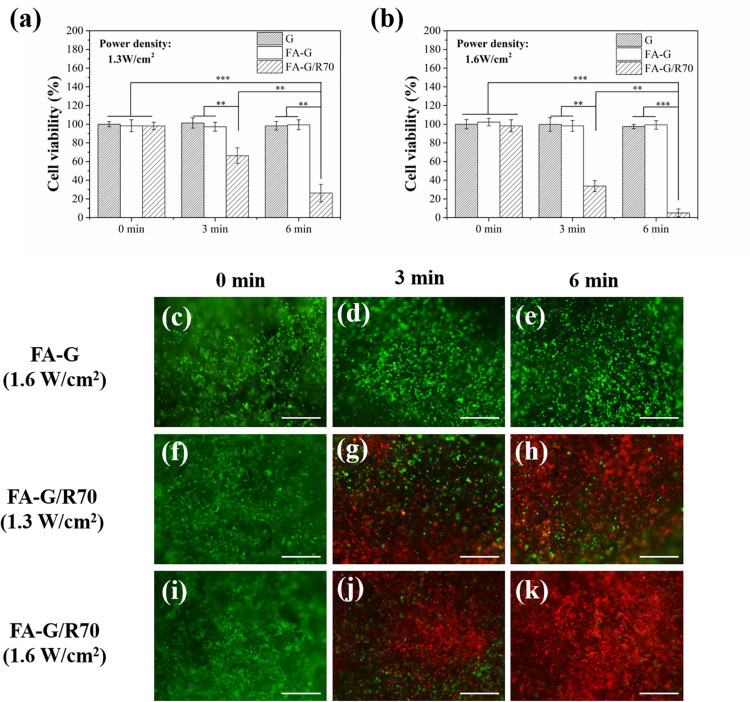
Cell viability of MDA-MB231 cells in the G, FA-G, and FA-G/R70 scaffolds with different laser irradiation for 0, 3, and 6 min. **(a)** 1.3 W/cm^2^ laser power intensity, **(b)** 1.6 W/cm^2^ laser power intensity. **(c–k)** Live/dead staining of MDA-MB231 cells in FA-G and FA-G/AuNRs70 with laser irradiation for 0, 3, and 6 min. Scale bar: 100 μm. Data represent means ± SD (*n* = 3). **P < 0.01; ***P < 0.001.

MDA-MB231 cells were used for investigating *in vitro* photothermal ablation effect of the composite scaffold FA-G/R70. After cells were cultured in composite scaffolds for 1 day, the cell/scaffold constructs were irradiated with NIR laser. Two power densities of NIR laser were used for the irradiation. Cell viability before (0 min) and after irradiation (3 and 6 min) was measured (Figures 5a,b). The cells showed high cell viability both in FA-functionalized gelatin scaffold and FA-functionalized composite scaffold before laser irradiation. After irradiation with NIR laser, cell viability decreased. When irradiation time increased, cell viability in FA-G/R70 scaffold significantly decreased to 66.3% after 3-min and to 26.3% after 6-min irradiation with 1.3-W/cm^2^ laser. When laser power intensity increased to 1.6 W/cm^2^, cell viability decreased more rapidly than that irradiated with 1.3 W/cm^2^. Cell viability decreased to 33.7% after 3-min and to 4.3% after 6-min irradiation with 1.6-W/cm^2^ NIR laser.

Live/dead staining further demonstrated the hyperthermal ablation effect of the composite scaffolds (Figures 5c–k). After NIR laser irradiation, the live cells were stained in green fluorescence, while dead cells in red fluorescence. Before NIR laser irradiation, almost all the MDA-MB231 cells were alive. However, after NIR irradiation, some of the cells were dead. The number of dead cells increased with laser power intensity and irradiation time. Under irradiation of a power intensity of 1.6 W/cm^2^ for 6 min, almost all the cells were dead (Figure 5k). For control scaffold without AuNPs, no dead cells were observed even after NIR laser irradiation (Figures 5d,e). All the results indicated that the FA-G/R70 scaffold had a high photothermal ablation effect. The high ablation effect of the FA-G/R70 scaffold was due to its excellent PTT conversion property at the NIR laser irradiation wavelength. Cancer cell can be killed when temperature is higher than 42°C (Beqa et al., 2011). As shown in Figure 3B, when the FA-G/R70 scaffold was irradiated with NIR laser at a power intensity of 1.3 W/cm^2^ for 3 and 6 min or 1.6 W/cm^2^ for 3 and 6 min, the scaffold temperature increased 25.7, 28.6, 33.9, and 35.1°C over room temperature, respectively. The temperature after NIR laser irradiation was much higher than the tolerance temperature of the cancer cells, subsequently killing the cells.

### *In vivo* Photothermal Ablation of Cancer Cells by FA-Functionalized Gelatin–AuNPs Composite Scaffolds

**FIGURE 6:**
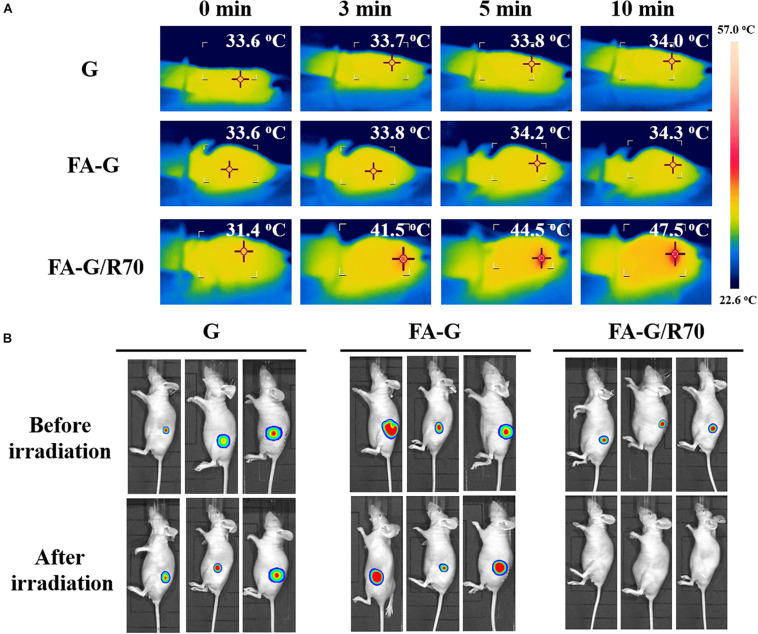
Temperature monitoring of the implantation sites during NIR laser irradiation and bioluminescence imaging of mice before and after NIR laser irradiation. **(A)** Temperature monitoring of the implanted MB231-Luc cells/scaffold constructs of G, FA-G, and FA-G/R70 scaffolds during NIR laser irradiation at a power intensity of 1.6 W/cm^2^ for 10 min. **(B)** Whole-body bioluminescence imaging of mice subcutaneously implanted with MB231-Luc cells/scaffold constructs of G, FA-G, and FA-G/R70 scaffolds before and after NIR laser irradiation.

After confirmation of the *in vitro* photothermal ablation effect the FA-G/R70 scaffold, animal experiment was designed to further confirm its *in vivo* photothermal ablation property. The MDA-MB231-Luc cells cultured in the FA-G/R70 scaffold for 3 days, and then the cells/scaffold constructs were subcutaneously implanted in nude mice. After 3 days’ implantation, the implantation sites were irradiated with NIR laser for 10 min. Gelatin and FA-functionalized gelatin scaffolds were used as controls. Temperature monitoring during NIR laser irradiation showed that the local temperature of the control G and FA-G scaffolds was kept almost unchanged during NIR laser irradiation (Figure 6A). However, the local temperature of the implantation sites of cells/FA-G/R70 constructs increased to 41.5, 44.5, and 47.5°C after irradiation for 3, 5, and 10 min, respectively, (Figure 6A). Whole-body bioluminescence imaging showed the *in vivo* photothermal ablation effect of the composite scaffolds (Figure 6B). All the mice implanted with the FA-G/R70 composite scaffold and G and FA-G control scaffolds showed strong luminescence imaging before NIR laser irradiation. The result indicated that the MDA-MB231-Luc cells were alive and survived after subcutaneous implantation in nude mice. After NIR laser irradiation for 10 min, the mice implanted with G and FA-G control scaffolds still showed strong luminescence, suggesting the MDA-MB231-Luc cells were still alive, not killed. On the other hand, no luminescence was detected in the mouse implanted with the FA-G/R70 composite scaffold after NIR laser irradiation. These results indicated that the FA-G/R70 composite scaffold could kill all the cancer cells under NIR laser irradiation because of its photothermal property. For future practical application in clinics, the composite scaffolds can be implanted after surgical resection of breast cancers. The composite scaffolds can be expected to target and capture remaining cancer cells and ablate them by repeating hyperthermal effect through laser irradiation. Therapeutic drugs can also be introduced in the composite scaffolds to realize combination of PTT and chemical therapy.

## Conclusion

FA-functionalized gelatin–AuNPs composite scaffolds were prepared by hybridizing FA-conjugated gelatin and FA-modified AuNPs and by using ice particulates as a porogen material. The composite scaffolds had the interconnected pore structures with large spherical pores surrounded with small pores. The composted scaffolds showed good photothermal properties and capture capacity for FA receptor–positive cancer cells. Temperature change of the composite scaffolds under NIR laser irradiation increased with laser power intensity and irradiation time. The FA-G/R70 composite scaffold showed the best photothermal property. *In vitro* cell culture and *in vivo* animal experiments demonstrated that the FA-G/R70 composite scaffold could kill cancers under NIR laser irradiation. The FA-functionalized gelatin–AuNPs scaffolds should be useful for local photothermal ablation of breast cancer cells for breast cancer therapy.

## Data Availability Statement

All datasets presented in this study are included in the article/Supplementary Material.

## Ethics Statement

The animal study was reviewed and approved by Animal Experiments Committee of National Institute for Materials Science.

## Author Contributions

GC, NK, and YY designed the research. HC, XW, LS, and TZ did the experiments and analyzed experimental data. NK did the data analysis and figure revision. HC, YY, and GC wrote the manuscript. All authors contributed to the article and approved the submitted version.

## Conflict of Interest

The authors declare that the research was conducted in the absence of any commercial or financial relationships that could be construed as a potential conflict of interest.
