# Supplementary material for: Folic Acid–Functionalized Composite Scaffolds of Gelatin and Gold Nanoparticles for Photothermal Ablation of Breast Cancer Cells
Source: Front Bioeng Biotechnol. 2020 Nov 4;8:589905. doi: 10.3389/fbioe.2020.589905 (PMC7671968; doi:10.3389/fbioe.2020.589905)
Supplement: Supplementary file 1 [file Data_Sheet_1.PDF]

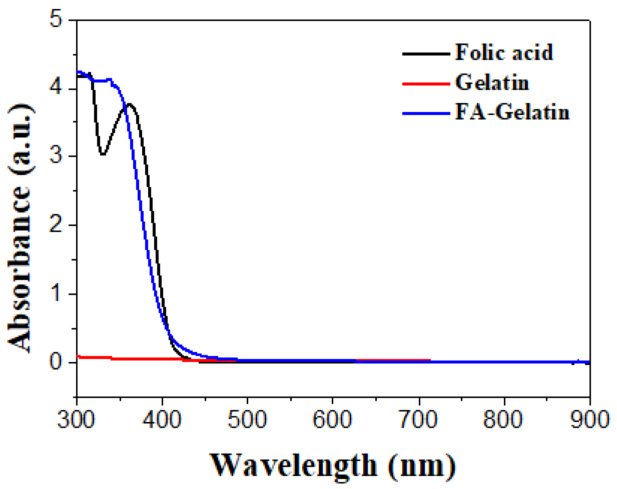

Figure S1 UV-Vis spectrum of folic acid, gelatin and FA-gelatin

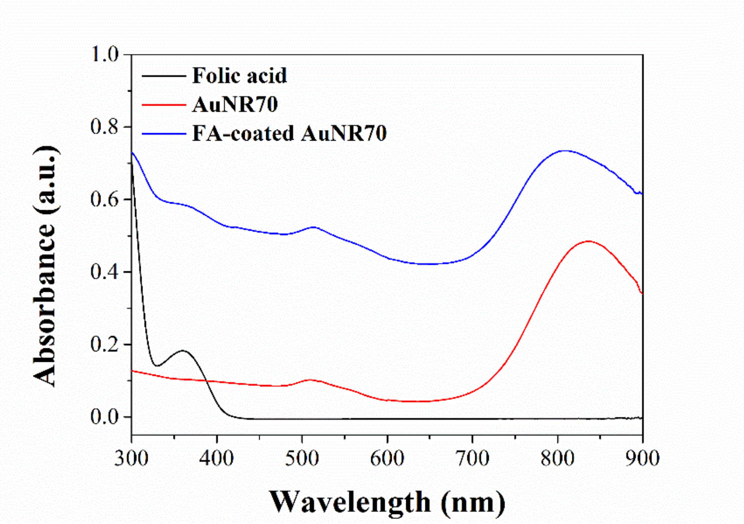

Figure S2 UV-Vis spectrum of folic acid, AuNR70 and FA-coated AuNR70
